# Predictive validity of the 5-item Compliance Questionnaire for Rheumatology (CQR5) in detecting poor adherence of patients with rheumatoid arthritis to biological medication

**DOI:** 10.1186/s13075-020-02319-4

**Published:** 2020-09-29

**Authors:** Fausto Salaffi, Marco Di Carlo, Marina Carotti, Luca Ceccarelli, Sonia Farah, Daniela Marotto, Valeria Giorgi, Piercarlo Sarzi-Puttini

**Affiliations:** 1 Rheumatology Clinic, Dipartimento di Scienze Cliniche e Molecolari, Università Politecnica delle Marche, “Carlo Urbani” Hospital, Via Aldo Moro 25, 60035 Jesi, Ancona Italy; 2 Dipartimento di Scienze Radiologiche S.O.D. Radiologia Pediatrica e Specialistica, Azienda Ospedaliera Universitaria, Ospedali Riuniti “Umberto I – G.M. Lancisi – G. Salesi”, Ancona, Italy; 3 Divisione di Reumatologia, Dipartimento di Medicina Interna, ASST Fatebenefratelli-Sacco, Milan University School of Medicine, Milan, Italy

**Keywords:** Rheumatoid arthritis, Adherence, CQR5, Biological disease-modifying anti-rheumatic drugs

## Abstract

**Background:**

Adherence is a key factor for therapeutic success in patients with rheumatoid arthritis (RA). The aim of this study was to determine whether results from the 5-item Compliance Questionnaire for Rheumatology (CQR5) can predict future poor adherence to biological disease-modifying anti-rheumatic drugs (bDMARDs) in patients with RA, using medication possession ratio (MPR) as the gold standard comparator.

**Methods:**

RA patients starting a bDMARD were prospectively followed for 12 months. At baseline, CQR5 was collected in relation to the prescribed bDMARD. Patients were dichotomised into good adherers and poor adherers, categories that were then used as the variable in a predictive function analysis of the CQR5 in order to determine the accuracy of the classification at the end of the study period in comparison with the MPR. The sensitivity, specificity, and likelihood ratio of detecting poor adherers were also determined because this is the clinically important purpose of the questionnaire. Satisfactory adherence was defined as > 80% compliance with the prescribed dose regimen.

**Results:**

Of the 210 RA patients enrolled (147 women and 63 men; mean age 58.6 ± 12.8 years; mean disease duration 7.4 ± 2.5 years), at the end of the 12-month follow-up, 152 patients (72.4%) were good adherers and 58 (27.6%) were poor adherers according to MPR. Predictive analyses showed that the sensitivity and specificity of the CQR5 in detecting poor adherence were respectively 89.9% (95% CI 84.07–94.10%) and 80.8% (95% CI 67.46–90.37%). The accuracy of the CQR5 was 83.04% (95% CI 77.27–87.85%), the positive likelihood ratio (i.e. detecting ≤ 80% adherence) 4.67 (95% CI 2.58–8.18), and the area under curve 0.85 (95% CI 0.79–0.89).

**Conclusion:**

Higher baseline CQR5 scores significantly predict the treatment adherence of RA patients. This suggests that this instrument could be used for screening purposes in order to identify patients who are poorly adherent to bDMARDs.

## Introduction

Treatment adherence is the extent to which patient behaviour coincided with healthcare [1] and, unlike compliance (which refers to a physician’s treatment plan), indicates a patient’s active and voluntary role in accepting the prescribed therapy and therapeutic schedule, and respecting correct daily dosing over time, whereas treatment persistence describes the continuation of treatment for the prescribed period [2, 3]. Non-adherence to treatment often explains a failure to achieve therapeutic goals in rheumatoid arthritis (RA) patients [4].

One significant aspect of the strategies to improve medication adherence is to understand its magnitude. However, there is a lack of general guidance for researchers and healthcare professionals to choose the appropriate tools that can explore the extent of medication adherence and the reasons behind this problem, in order to orchestrate subsequent interventions.

Adherence to disease-modifying anti-rheumatic drug (DMARD) prescriptions has been highly variable in clinical trials [5], and retrospective studies of treatment persistence in RA patients taking tumour necrosis factor alpha (TNFα) antagonists have shown that is 82–89% after 6 months, 48–78% after 12 months, 70% after 13 months, 71% after 18 months, and 62–67% after 24 months [6–8]. Bluett et al. found that 27% of the patients in a UK study reported non-adherent behaviours during the first 6 months of treatment with a biological drug [9]. The multi-centre, non-interventional, retrospective Study on Adherence of Rheumatoid Arthritis patients to Subcutaneous and Oral Drugs (ARCO) involving 42 Spanish rheumatology clinics found non-adherence to the prescribed biological drug in 14.3% of patients [10], and an ARCO substudy found non-adherence in 20.9% [11]. Our own group’s previous study found that 20.6–21.7% of patients subcutaneously treated with anti-TNFα agents were non-adherent [4, 12], and randomised clinical trials of various TNFα antagonists have found adherence rates of 73–86% after 13.5 months and 55–68% after 25.5 months [13, 14].

There are numerous tools for measuring medication adherence, but these need to prove to be valid, reliable, and sensitive to change [15]. Generally, measurements of medication adherence are categorised by the World Health Organization (WHO) as subjective and objective measurements [16]. Subjective measurements involve those requiring provider’s or patient’s evaluation of their medication-taking behaviour. Self-report and healthcare professional assessments are the most frequently used tools [17]. Objective measures include pill counts, electronic monitoring such as the Medication Events Monitoring System (MEMS), secondary database analysis such as the medication possession ratio (MPR), and biochemical measures [18]. These objective measures represent an improvement over subjective measures [17].

The most frequently used measures in large-scale clinical trials are self-assessment questionnaires. With them, attitudes and intentions can be evaluated, helping to understand the reasons for non-adherence, and measuring non-adherence to a specific drug regimen in a standardised way [19]. Self-report questionnaires, which have a reasonable predictive power, are more useful in a busy, resource-limited clinical setting with moderate to high literacy population [20].

The validated 5-item Compliance Questionnaire for Rheumatology (CQR5), in particular, has proved to be reliable in distinguishing patients who are to be good adherers to anti-rheumatic treatment from those who are likely to be poor adherers [21].

The aim of this prospective longitudinal study was to assess the validity of the CQR5 in predicting poor adherence to biological DMARDs (bDMARDs) in patients with RA, using MPR as gold standard comparator.

## Methods

### Study population

This prospective longitudinal observational cohort study included adult RA patients, diagnosed according to the 2010 American College of Rheumatology/European League Against Rheumatism classification criteria [22], from December 2017 to March 2020. Patients with at least moderate disease activity, defined by a Clinical Disease Activity Index (CDAI) > 22, to whom a subcutaneously administered bDMARD was introduced in therapy for the first time or who made a switch were included.

The exclusion criteria were any inflammatory or rheumatic disease other than RA, severe ongoing infections, hypersensitivity to the active substance or any of the excipients, and pregnancy.

### Study protocol and data collection

All of the patients completed a comprehensive questionnaire covering socio-demographic and disease-related variables: age, sex, disease duration (defined as the time since diagnosis), educational level (primary, secondary school, and university), disease activity, physical function, drugs, frailty, radiographic damage, and adherence testing. The laboratory tests and clinical assessments considered the presence of rheumatoid factor (RF), the erythrocyte sedimentation rate (ESR), the 28-joint swollen and tender joint counts (SJC and TJC), and the patient and physician global assessments of RA activity (PtGA and PhGA) on 0–10 numerical rating scales (NRS). These clinical variables were used to calculate the CDAI, the only composite index that does not incorporate an acute phase response and can therefore be used to evaluate disease activity anytime and anywhere. CDAI ranges from 0 (totally inactive disease) to 76 (very active disease), and CDAI of ≤ 2.8 corresponds to remission [23]*.*

#### Comorbidities

The comorbidities were assessed using the modified Rheumatic Disease Comorbidity Index (mRDCI) [24, 25]. The formula for calculating the mRDCI is: 1* lung disease and [2* (myocardial infarction, other cardiovascular diseases, or stroke) or 1* hypertension] and 1* (ulcer or other gastrointestinal diseases) and 2* kidney disease and 1* if BMI is > 30 or 2* if BMI is > 35, and 1 for each of diabetes, fracture, depression, and cancer [25].

#### Functional ability

The Health Assessment Questionnaire Disability Index (HAQ-DI) evaluates the difficulty of carrying out everyday living activities in eight domains. The final HAQ-DI score ranges from 0 to 3, with higher scores indicating greater disability [26].

#### Frailty

Frailty was evaluated using the Comprehensive Rheumatologic Assessment of Frailty (CRAF) [27], a recently validated multidimensional index that does not require a calculator. It is defined by investigating 10 health domains: nutritional status, weakness, falls, comorbidities, polypharmacy, social activity, pain, fatigue, physical function, and depression. Weakness was assessed testing handgrip strength (HGS), estimated using a cylinder-shaped electronic device with five sensors [28, 29]. The ten scores were added and divided by the total number of deficits evaluated to produce a CRAF index between 0.0 and 1.0. The CRAF cut-off points have been established using Clegg’s criteria [30] as follows: score ≤ 0.12 represents patients without frailty, score > 0.12 and ≤ 0.24 represents patients with mild frailty, score > 0.24 and ≤ 0.36 represents patients with moderate frailty, and score > 0.36 represents patients with severe frailty.

#### Adherence

Adherence was assessed at the beginning of bDMARD treatment using the validated Italian CQR5 [31], and at the end of the 12 months of follow-up using the MPR [32, 33]. The development of the CQR5 has been previously described in detail [17]. The responses to the CQR5 questions are semi-quantitative and based on a 4-point Likert-like scale ranging from “definitely do not agree” (scored 1) to “definitely agree” (scored 4), with lower scores indicating less adherence. The CQR5 was developed after factor analysis of the 19-item CQR (CQR19) [21, 34] and, like the original, identifies poor adherers (i.e. patients taking < 80% of their medication correctly) and good adherers (i.e. patients taking ≥ 80% of their medication correctly) [34]. The CQR19 has been validated in patients with inflammatory rheumatic diseases against a MEMS. The CQR19 compared well with electronic monitoring over 6 months with a sensitivity of 98%, a specificity of 67%, and an estimated kappa of 0.78 to detect non-adherence [35].

The reduced number of items of the CQR5 makes it useful for screening and monitoring purposes in clinical practice [21]. CQR5 increases the clinical utility by diminishing the patient burden. Like the original CQR19, CQR5 identifies poor adherers and good adherers [31].

At the end of the study, the MPR of each patient was calculated using the following formula: MPR = (the number of days actually covered by the medication/the number of days theoretically covered by the medication) × 100. The MPR takes on positive numbers including zero. MPR of zero means no adherence, while an MPR of one means perfect adherence. MPR measures the percentage of time a patient has access to medication [32]. The MPR is ubiquitously used throughout the healthcare industry [32, 33], and a patient is considered a PA if the MPR is ≤ 80%. The first day of the study period was the day on which the patient was administered the first injection of the prescribed bDMARD, and the last day was the day before the first injection outside the study period. In the event that a patient discontinued the administration of the bDMARD because of incident infection, surgery, or any other reason, the number of days of discontinuation was subtracted from the duration of the study period in order to obtain the actual number of days that should have been covered by the prescribed medication. Differences in dosing regimens and periods of discontinuation meant that the length of the study period may have varied from patient to patient.

### Statistical analysis

The data were analysed using MedCalc®, version 19.0.1.0 (MedCalc Software, Mariakerke, Belgium). Continuous data are presented as mean values and standard deviations (SDs) or median values with interquartile ranges (IQRs) depending on their distribution, which was tested using the Kolmogorov-Smirnov test. The patients were classified into two categories: good adherers and poor adherers, according to MPR.

The *χ*^2^ test was used to compare categorical variables, and the Mann-Whitney *U* test for continuous variables. *p* values of ≤ 0.05 were considered statistically significant.

Predictive validity of CQR5 compared to MPR (external dichotomous criterion) in identifying adherence categories was analysed in terms of sensitivity, specificity, area under the curve (AUC), and positive likelihood ratio [36]. For all these variables, 95% confidence intervals (CI) have been considered. Predictive validity is a type of criterion validity that “refers to the ability of a measure to predict some subsequent and temporarily ordered criterion effectively” [37].

## Results

The study involved 210 RA patients: 147 women (70%) and 63 men (30%) with a mean age of 58.6 ± 12.8 years, a mean disease duration of 7.4 ± 2.5 years, and a mean BMI of 26.1 ± 4.0 kg/m^2^. Their mean CDAI, HAQ-DI, and mRDCI were respectively 26.6 ± 9.5, 1.2 ± 0.6, and 1.9 ± 2.0, and the most frequent comorbidities were hypertension (74 patients, 48.7%), metabolic disorders (48 patients, 31.6%), and endocrinological diseases (42 patients, 27.6%). All of the patients were receiving at least one bDMARD (adalimumab [85 patients, 40.5%], etanercept [80 patients, 38.1%], abatacept [28 patients, 13.3%], golimumab [10 patients, 4.7%], and tocilizumab [7 patients, 3.3%]), most of whom (73.8%) were on their first biological agent. Approximately 70% of the patients were also receiving a conventional synthetic DMARD such as methotrexate (67.1%) or hydroxychloroquine (15.7%), 49 (23.3%) were taking oral corticosteroids at a mean prednisone or equivalent dose of 6.2 mg/day (range 2.5–25), and 98 (46.6%) were receiving NSAIDs on demand. Table 1 summarises the demographic and clinical characteristics of the whole cohort.

**Table 1:** Baseline demographic, laboratory, and clinimetric data

	Mean	Median	SD	IQR
Age, years	58.68	56.00	12.80	48.00–70.00
Disease duration, years	7.44	7.10	2.87	5.00–10.00
Education, years	11.05	12.00	3.78	8.00–13.00
BMI (kg/m^2^)	26.2	4.18	24.32	23.66–29.21
mRDCI, range 0–11	1.94	1.00	2.03	0.00–3.00
ESR (mm/h), range 0–150	37.98	35.50	18.92	24.00–50.00
HAQ-DI, range 0–3	1.19	1.00	0.61	0.87–1.50
CDAI, range 0–76	26.66	26.00	9.51	21.00–33.00
CRAF, range 0–10	0.27	0.19	0.22	0.080–0.41

*SD* standard deviation, *IQR* interquartile range, *BMI* body mass index, *mRDCI* modified Rheumatic Disease Comorbidity Index, *HAQ-DI* Health Assessment Questionnaire Disability Index, *CDAI* Clinical Disease Activity Index, *CRAF* Comprehensive Rheumatologic Assessment of Frailty

The MPR at the end of the follow-up indicated that 152 of the 210 patients (72.4%) were good adherers and 58 (27.6%) poor adherers. The poor adherers had a higher age (*p* < 0.0001), higher CDAI (*p* < 0.0001), higher CRAF score (*p* = 0.0036), higher mRDCI (*p* = 0.0039), higher ESR (*p* = 0.0335), and higher HAQ-DI (*p* = 0.0336) (Table 2). The female population was less adherent than the male population (*p* = 0.0001).

**Table 2:** Baseline demographic, clinical, and laboratory variables of poor adherers versus good adherers

	Overall cohort of RA patients (*n* = 210)				
	Poor adherers (*n* = 58)		Good adherers (*n* = 152)		
	Median	IQR	Median	IQR	*p* value*
Age, years	61.00	50.00–75.00	55.00	48.00–69.00	< 0.0001
Disease duration, years	7.00	5.00–10.00	7.00	5.00–10.00	0.9034
Educational level, years	12.00	8.00–13.00	13.00	8.00–14.00	0.8284
BMI (kg/m^2^)	25.02	22.33–27.91	4.51	24.33–29.79	0.6041
mRDCI, range 0–11	1.50	0.50–3.50	1.00	0.00–3.00	0.0039
ERS (mm/h), range 0–150	39.50	27.50–54.00	34.50	22.00–49.00	0.0335
HAQ-DI, range 0–3	1.31	0.92–1.87	1.00	0.86–1.50	0.0336
CDAI, range 0–76	31.00	22.00–39.00	25.00	20.00–31.00	< 0.0001
CRAF, range 0–10	0.44	0.19–0.66	0.17	0.08–0.34	0.0036

*IQR* interquartile range, *BMI* body mass index, *mRDCI* modified Rheumatic Disease Comorbidity Index, *HAQ-DI* Health Assessment Questionnaire Disability Index, *CDAI* Clinical Disease Activity Index, *CRAF* Comprehensive Rheumatologic Assessment of Frailty

*Mann-Whitney *U* test

In comparison with the MPR, the classification table of the calculated discriminant ability of the CQR5 questionnaire to detect ≤ 80% treatment adherence showed a sensitivity and specificity of respectively 89.9% (95% CI 84.07–94.10%) and 80.8% (95% CI 67.46–90.37%), an accuracy of 83.04% (95% CI 77.27–87.85%), an AUC of 0.85 (95% CI 0.79–0.89), and a positive likelihood ratio of 4.67 (95% CI 2.58–8.18) (Table 3).

**Table 3:** Discriminant validity of the CQR5 to identify ≥ 80% adherence by MPR after 1 year of bDMARD treatment

Sensitivity	89.87%	84.07–94.10%
Specificity	80.76%	67.46–90.37%
Area under the curve	0.85	0.79–0.89
Positive likelihood ratio	4.67	2.67–8.17
Negative likelihood ratio	0.12	0.07–0.20
Positive predictive value	60.90%	47.09–73.16%
Negative predictive value	95.98%	93.65–97.48%
Accuracy	83.04%	77.27–87.85%

*CQR5* 5-item Compliance Questionnaire for Rheumatology, *MPR* medication possession ratio, *bDMARD* biological disease-modifying anti-rheumatic drug

## Discussion

In this study, we clearly demonstrated the validity of CQR5 as a predictor of poor adherence to bDMARD treatment in patients with RA.

The ABC taxonomy defines medication adherence as the process by which patients take their medication as prescribed [38]. Adherence to drug treatment is a challenge for many RA patients as increasing comorbidities and disability mean that they have to take an increasing number of drugs simultaneously, and poor adherence can lead to higher rates of hospitalisation and institutionalisation [33, 39].

Poor adherence not only has been shown to adversely affect the patient’s health, but also puts financial strain on the healthcare system [40]. As such, measuring adherence is a highly sought-after parameter in the health industry and has been proposed to be used for evaluating the quality of care. Furthermore, it has also been used to quantify the medication sales performance in the pharmaceutical industry.

There is no gold standard for the assessment of adherence. As a result, studies on adherence to conventional and bDMARDs in patients with RA have reported suboptimal adherence rates, although with highly variable results [10, 41]. A systematic review has found that the overall range of continuation to biologic treatments in patients with RA, at 12 months, was 32.0 to 90.9% depending on the definition of adherence, duration of follow-up, and method of measurement [42]. Waimann et al. found that adherence to oral DMARD and steroid treatment in patients with RA ranged from 58 to 71% [43] and that only 20% of the patients showed > 80% adherence. Bluett et al. found that 57 out of 286 patients (20%) did not adhere to biological treatment, and it has also been reported that 21–35% of patients administered anti-TNF agents discontinue treatment within the first year [9]. In line with previous findings [4–12], we found that 27.6% of patients treated with biological agents were non-adherers at the end of 12 months of follow-up.

Measuring the adherence of adult RA patients requires an adherence measurement system that appropriately considers chronic diseases and comorbidities, frailty, and the patients’ motor, sensory, and cognitive capacities within their community setting, as well as data management by healthcare providers. It must also be practical and allow patients to come to terms with the difficulties of simultaneously taking multiple drug therapies.

Medication adherence can be assessed using numerous subjective (e.g. self-reporting, physicians’ assessment), direct (e.g. biomarkers), or indirect (e.g. pharmacy refill, tablet counts, electronic monitors, questionnaires) measurements, each with potential advantages or disadvantages [32, 44]. Measurement of adherence in patients with RA can be complicated further in the case of bDMARDs because these compounds have dosing regimens and dosing frequencies that may change during treatment.

In a closed pharmacy system, prescription records can be used to calculate surrogate measures of adherence, such as MPR, which have been correlated with biological and clinical outcomes [1]. However, the calculation of pharmacy-based adherence measurements, such as MPR, is not feasible in many clinical settings. A sophisticated indirect adherence measurement is MEMS. MEMS is the electronic detection of drug intake through the presence of microcircuits inside the packaging. This sophisticated system detects and memorises the operations necessary to remove a dose of the drug. MEMS is currently considered the gold standard for measuring adherence, but it is also not feasible in routine practice in most settings [19].

Treatment persistence and adherence to biological regimens have been measured in many studies, but the value of adherence measurement system in predicting poor adherence to biological medications in patients with RA has not been investigated in detail. In our study, the CQR5 was chosen because it is the only validated adherence questionnaire in rheumatology [21, 31]. Self-reported adherence measures are easily administered but less sensitive than pharmacy-based measures and tend to overestimate true adherence. On the other hand, self-reported adherence measures are simple and correlated with rheumatological outcomes [4, 12]. Compared to other more intrusive measures, self-reported adherence measures are characterised by low costs, minimal participant burden, ease and administrative speed, and flexibility in terms of mode of administration and timing of assessment. A disadvantage of self-reported adherence measures could be represented by the overestimation of adherence compared to MEMS [45], yielding false negatives in the screening for poor adherence [46].

Our findings provide evidence for the utilisation of the CQR5 in routine clinical practice. In this study, we have identified the degree to which an adherence measurement system predicts adherence in relation to pharmacy-based measures. Predictive validity of an adherence measurement system like CQR5 is of particular interest to rheumatologists, since this measurement system can provide important information on future patient behaviour in relation to challenging therapies such as those with bDMARDs.

Our findings need to be interpreted taking into account some limitations of the study design. First of all, the relatively short duration of the study may have influenced the findings, which therefore cannot be extended to longer periods. Secondly, adherence may have been overestimated due to the Hawthorne effect. Patients were aware of being under observation, and this fact may have altered their behaviour and made them more conscientious in medication use.

## Conclusion

In this study, we demonstrated the predictive validity of CQR5 in identifying poor adherence to bDMARD treatment in RA patients. Our results suggest that CQR5 may be useful in clinical practice, given its association with MPR. In particular, any self-reported non-adherence is an important clinical indicator that should prompt education, counselling, and adherence intervention.

## Data Availability

The datasets used and/or analysed during the current study are available from the corresponding author on reasonable request.
